# Dental anxiety and oral health in American Indian and Alaska natives

**DOI:** 10.1111/jphd.12633

**Published:** 2024-07-02

**Authors:** Tamanna Tiwari, Casey D. Wright, Lisa J. Heaton, Morgan Santoro, Eric P. Tranby

**Affiliations:** ^1^ School of Dental Medicine University of Colorado Aurora Colorado USA; ^2^ Department of Developmental Sciences Marquette University School of Dentistry Milwaukee Wisconsin USA; ^3^ Analytics and Data Insights CareQuest Institute for Oral Health Boston Massachusetts USA

**Keywords:** American Indian/Alaska native, dental anxiety, dental trust

## Abstract

**Objective:**

American Indian and Alaska native (AI/AN) individuals report distrust of the healthcare system. This study explored associations between having either high levels of dental distrust or high levels of dental care‐related fear and anxiety (“dental anxiety”) and oral health outcomes in AI/AN adults.

**Methods:**

The 2022 State of Oral Health Equity in America survey included the Modified Dental Anxiety Scale and asked to what extent respondents agreed with the statement, “At my last oral health visit, I trusted the oral health provider I saw”, and asked about self‐rated oral health and presence of a dental home.

**Results:**

AI/AN individuals (*N* = 564) who reported low dental trust (*n* = 110) or with high dental anxiety (MDAS≥19; *n* = 113) reported significantly worse overall and oral health and were significantly less likely to have a dental home (*p* < 0.05 used for each analysis).

**Conclusion:**

Dental distrust and dental anxiety can significantly impact oral health and dental utilization in AI/AN communities and are important intervention targets to improve AI/AN oral health.

## INTRODUCTION

American Indian and Alaska native (AI/AN) populations have the highest burden of oral diseases, including periodontal disease and tooth loss, among all racial and ethnic minority populations in the US [1]. AI/AN individuals face greater social, physical, economic, emotional, and environmental barriers in accessing healthcare overall, leading to poor health outcomes and decreased quality of life [2]. The federal government has a “trust responsibility” based on several treaties and legislative acts to protect tribal land, assets, and recourses and provide health care for AI/AN, and the Indian Health Service (IHS) continues to meet this responsibility [3]. The IHS, however, faces shortfalls in funding and staff, which creates other potential obstacles for AI/AN individuals [3]. Furthermore, AI/AN populations have reported perceived discrimination and racism as barriers to healthcare utilization and distrust of healthcare providers and the system [4]. However, no known literature has examined the AI/AN population's trust in dental providers.

Cultural, historical, and psychosocial stress have been reported in AI/AN communities at a much higher level compared with the general US population, as have depression and anxiety [5]. In the general population, dental care‐related fear and anxiety (hereafter noted as simply “dental anxiety”) is associated with delaying or avoiding dental treatment and can lead to poor oral health and greater untreated dental diseases [6]. Dental anxiety in rural and isolated populations has previously been shown to be negatively correlated with oral health‐related quality of life [7, 8]. Approximately 22% of AI/AN individuals live on a reservation or trust land, which are often in rural and isolated areas—suggesting a potentially similar association.

Members of other ethnic minority groups, such as Hispanic populations, avoid dental visits due to dental care related fear and anxiety, but no known studies have directly explored dental fear and anxiety among AI/AN populations [9]. Moreover, a dearth of literature exists more generally regarding dental anxiety in the AI/AN population, except for one recent white paper report by CareQuest Institute for Oral Health [10]. There, a significantly higher proportion of the AI/AN individuals demonstrated greater dental anxiety when compared with those who did not identify as AI/AN [10]. The previously published white paper report provided historical context, data on overall oral health disparities, and health care delivery models in AI/AN communities [10].

This brief report was aimed at beginning to address the gap in the literature regarding dental trust and dental utilization among the AI/AN population. In addition, this study explores reports of dental anxiety in AI/AN adults, shedding light on the correlation of dental anxiety with oral health status and dental care utilization.

## METHODS

This study used data from the 2022 State of Oral Health Equity in America (SOHEA) survey that assessed adult consumers' attitudes, knowledge, and experiences with oral and overall health care. CareQuest Institute for Oral Health designed the survey, and data were collected by the National Opinion Research Center at the University of Chicago in January–February 2022 from adults 18 years and older from the AmeriSpeak® panel. AmeriSpeak is a probability‐based panel designed to be representative of the US household population. Randomly selected US households were sampled using area probability and address‐based sampling, with a known, nonzero probability of selection from the NORC National Sample Frame. Sampled households were contacted by US mail, telephone, and field interviewers. A sampling unit of 17,603 was used, with a final sample size of 5682 and a final weighted cumulative response rate of 4.0%. All data presented account for appropriate sample weights, and the margin of error for the survey was 1.75%.

An additional nonprobability, opt‐in sample of AI/AN individuals was recruited for the 2022 round of data collection for a total of 564 AI/AN participants (9.9% of the total sample; see Table 1). Just under half (48.2%) of the AI/AN participants were recruited through the AmeriSpeak® probability panel, and 51.8% came from an additional opt‐in nonprobability online sample (Lucid Holdings, Inc.) for the sake of oversampling. AI/AN participants were offered the cash equivalent of $6 for completing the survey [6].

**TABLE 1:** Demographic characteristics (weighted percentages) of the American Indian/Alaska native (AI/AN) sample (from CareQuest Institute for Oral Health, 2023).

Sample	Opt‐in nonprobability sample	AmeriSpeak probability sample	AI/AN total sample
Totals	282	282	564
Race/ethnicity			
White, nonHispanic	0	0	0
Black, nonHispanic	0	0	0
Asian, nonHispanic	0	0	0
Other, nonHispanic	168 (48.5%)	66 (22.7%)	234 (36.0%)
2+ races, nonHispanic	60 (36.6%)	41 (24.2%)	101 (30.6%)
Age group			
18–29	94 (34.4%)	27 (15.2%)	121 (25.1%)
30–44	106 (33.7%)	77 (24.4%)	183 (29.2%)
45–59	56 (20.8%)	71 (25.5%)	127 (23.0%)
60+	26 (11.1%)	107 (34.9%)	133 (22.6%)
Gender			
Female	141 (47.6%)	149 (51.6%)	290 (49.5%)
Male	131 (48.8%)	129 (46.4%)	260 (47.6%)
Transgender	4 (1.8%)	1 (0.5%)	5 (1.2%)
Does not identify as male, female, or transgender	5 (1.7%)	3 (1.5%)	8 (1.6%)
Education			
Less than high school	22 (17.5%)	19 (14.3%)	41 (15.9%)
High school graduate or equivalent	92 (37.4%)	31 (24.6%)	123 (31.3%)
Vocational/tech school/associate	120 (31.1%)	141 (34.8%)	261 (32.9%)
Bachelor's degree	38 (9.7%)	50 (14.0%)	88 (11.8%)
Post grad study/professional degree	10 (4.2%)	41 (10.7%)	51 (8.1%)

Regarding outcome variables, survey respondents reported the recency of their last dental visit (either within the last year or more than a year ago), as well as whether or not they had a dental home, defined as “a single dentist or dental office that is your usual source of dental care.” Respondents rated their physical health, oral health (“state of your teeth, mouth, and gums”), and their “mental or emotional health” in three separate questions using a scale of “excellent/very good/good” or “fair/poor.”

To assess for provider trust, respondents indicated their level of agreement ranging from “strongly disagree,” “disagree,” “neither” agree nor disagree, “agree,” or “strongly agree” with the statement, “At my last oral health visit, I trusted the oral health provider I saw.” Responses were dichotomized as either “distrust” (0; strongly disagree, disagree, or neither) or “trust” (1; strongly agree or agree). Dental distrust included the response of “neither” along with strongly disagreeing or disagreeing as it was determined that individuals who trusted their oral health care provider would respond “strongly agree” or “agree,” and the “neither” option likely represented more disagreement than agreement with the statement.

The Modified Dental Anxiety Scale (MDAS) also was included in the SOHEA survey. The MDAS is a 5‐item measure that asks respondents to rate their anticipated level of anxiety (1 = not at all; 5 = extremely) across 5 common dental care scenarios, from having their teeth scaled and polished to having an intraoral injection [11]. In this study high dental anxiety was categorized by an MDAS score of 19 and higher; lower dental anxiety was defined as an MDAS score of 18 or lower [11]. As only 31 AI/AN individuals reported both higher dental anxiety and higher dental distrust, we examined these variables separately. Chi‐square analyses were used to test differences in oral health outcomes between AI/AN individuals who reported low dental anxiety versus high dental anxiety.

## RESULTS

General demographic information and descriptive statistics are included in Table 1.

### Dental distrust

Nearly one in five (19.5%, *N* = 110) AI/AN adults reported dental distrust. Compared to those that were trusting of providers, a greater proportion of AI/AN respondents who did not trust their dental provider had also not visited a dental provider in more than a year (71.4% vs. 41.7%, *p* < 0.05). More AI/AN respondents who trusted their dental provider identified with having a dental home (79.0%) compared to AI/AN respondents who did not trust their dental provider (42.1%, *p* < 0.01). A greater proportion of AI/AN respondents who did not trust their dental provider responded that their oral health was fair/poor (57.1%) compared to the proportion of AI/AN respondents who trusted their dental provider (32.1%, *p* = *p* < 0.05). This pattern was the same for those that endorsed fair/poor mental or emotional health (39.8% vs. 21.6% *p* < 0.05) and overall health status (35.8% vs. 19% *p* < 0.05).

### Dental anxiety

One in five (20.0%, *N* = 113) of AI/AN adults scored 19 or higher on the MDAS, indicating high levels of dental anxiety. Compared to those with less dental anxiety, AI/AN adults with greater dental anxiety were more likely to report not seeing a dentist in more than a year (45.2% vs. 65.0%, respectively). A greater proportion of AI/AN respondents with less dental anxiety reported having a dental home (77.2%) compared to AI/AN respondents with higher dental anxiety (50.0%, *p* < 0.05). A greater proportion of AI/AN respondents with high levels of dental anxiety responded that their oral health was fair/poor (60.0%) compared to the proportion of AI/AN respondents with lower dental anxiety (31.0%, *p* < 0.05). This pattern was the same for endorsing fair or poor mental/emotional health (45.0% vs. 20.7%, *p* < 0.05) and overall health (42.1% vs. 18.1%, *p* < 0.05).

## DISCUSSION

This study explored associations and differences among dental distrust, dental anxiety, and oral health outcomes among AI/AN individuals. Those from the AI/AN communities with less trust in dental providers or greater levels of dental care‐related fear and anxiety demonstrated significant differences in self‐perceptions of overall health and oral health, in dental utilization, and in having a dental home.

Results from this study support prior works, such that, mistrust of providers among minority populations was associated with poorer oral health outcomes [9]. Similarly, those with less provider trust indicated less utilization of dental services within the last year, which could perpetuate extant oral health problems. There is robust evidence demonstrating the impact of dental anxiety in general populations as it relates to avoiding care, resulting in overall distress, and poorer oral health outcomes [12]. In this study, those individuals of AI/AN communities with elevated dental anxiety likewise had poorer perceived overall health along with poorer perceived mental and emotional health. Mental health concerns, especially anxiety, are often comorbid with other mental health diagnoses or issues [12]. AI/AN individuals face greater mental health concerns and this could lead to overall anxiety about the dentist [13]. Similarly, dental anxiety can lead to avoidance of care, poorer oral health and self‐esteem, which also can impact overall emotional health. Given the scarcity of data in this area beyond this brief report, additional work is needed to help explore the full impact of dental fear and anxiety on overall well‐being, particularly among AI/AN individuals.

Similar to findings with lower dental provider trust, AI/AN adults with lower levels of dental anxiety were more likely to report having a dental home, whereas adults with high levels of dental anxiety did not. Overall, future work to understand dental provider trust and dental anxiety's association with dental care utilization should include the concept of provider cultural humility as a potential mechanism. Cultural humility is a commitment and active engagement in a lifelong process to educate healthcare providers to work with the increasing cultural, racial, and ethnic diversity as opposed to cultural competence that can potentially promote stereotyping of vulnerable populations [14]. Previous research has shown that AI/AN culture is misunderstood by many healthcare providers, and that can impact trust [15]. Additional research must examine the potential of historical trauma, structural racism, and other socio‐cultural roots of dental anxiety and distrust of dental providers and how cultural humility training can help dental providers in dental trust [16]. IHS clinics provide care to approximately 2.2 million of the 3.7 million AI/AN individuals living in the US [17]. Therefore, many AI/AN individuals may receive care from dental providers who are not aware of the unique historical factors that can impact trust in the dental setting. Other psychosocial factors, such as fear of pain, will be important to include as variables in future work in this area. Altogether, these connections between dental fear and anxiety, oral health, and emotional health only emphasize the need for research and implementation of training for dental providers to be cognizant of the emotional needs and be skilled in delivering trauma‐informed care to individuals in AI/AN communities and others experiencing historical trauma [18].

This study is not without its limitations. The sample of AI/AN respondents was small, which limits the generalizability to the greater AI/AN population. The survey design provided only limited possible race response options and did not include questions regarding respondents' tribal affiliations. There are hundreds of nations within the broad AI/AN community, and while understanding the nuances that each tribe faces is challenging, there may be some commonalities [19]. However, this study is a positive start to better understand the impact of dental provider trust and dental anxiety in the AI/AN community. Future studies can target more specific clinical data or other dental patient‐reported outcomes.

## CONFLICT OF INTEREST STATEMENT

Authors have no conflicts to report.
